# Neutrophils: emerging perspectives on the pathogenesis of oral mucosal diseases

**DOI:** 10.3389/fcimb.2025.1689266

**Published:** 2025-12-12

**Authors:** Yuhe Chen, Qian Mi, Fang Jia, Wenxia Meng

**Affiliations:** Departments of Oral Medicine, Stomatological Hospital, School of Stomatology, Southern Medical University, Guangzhou, Guangdong, China

**Keywords:** neutrophils, oral mucosal diseases, immune barrier, immune regulation, oral microenvironment

## Abstract

Neutrophils, as the predominant effector cells of innate immunity and the first line of defense at the oral mucosal barrier, play pivotal yet complex roles in both immune defense and pathological damage within the unique oral environment. The oral mucosa, continuously exposed to physical, chemical, and dense microbial challenges, necessitates a dynamic and robust immune response. Recent evidence highlights that neutrophils are not merely transient defenders but key regulators whose functions are profoundly shaped by the specific milieu of the oral cavity, differing significantly from other mucosal sites. However, the precise mechanisms by which neutrophils contribute to the pathogenesis of various oral mucosal diseases (OMDs), beyond their established role in barrier defense, remain inadequately synthesized and understood. This article systematically reviews the mechanisms underpinning neutrophil-mediated immune defense in the oral mucosa and critically summarizes their documented involvement in major OMDs, including oral candidiasis, recurrent aphthous ulcer, Behçet’s disease, and oral potentially malignant disorders/cancer. By elucidating the unique interplay between oral environmental cues (microbiota, function, systemic factors) and neutrophil behavior, this review aims to provide novel insights into the immunopathogenesis of OMDs and inform future translational research targeting neutrophil function.

## 1 Introduction

Neutrophils are polymorphonuclear cells (PMNs), the most abundant white blood cells and our body’s first-line immune defenders. Their life cycle involves rapid release from bone marrow, migration guided by infection signals, and pathogen clearance at the site via key mechanisms: phagocytosis, antimicrobial protein release (degranulation), reactive oxygen species (ROS) generation, and neutrophil extracellular trap (NET) formation – crucial for eliminating threats and starting inflammation resolution (Herro and Grimes, 2024).While traditionally thought to live only 6–8 hours, recent studies show inflammation can extend their circulation beyond 5 days and tissue persistence for weeks (Kraus and Gruber, 2021). These longer-lived (“senescent”) neutrophils also help regulate immune responses during inflammation’s resolution phase (Hsu et al., 2025). This expands our view of neutrophils from simple defenders to key players in maintaining overall body integrity, even without active infection.

These notions have led to widespread reports of neutrophils in mucosal organs. In mucosal organs such as the gastrointestinal tract and lungs, research has unequivocally demonstrated that neutrophils are key drivers of pathogenesis, leading to tissue damage and immune dysregulation through mechanisms such as excessive NETosis and ROS production (Eaton et al., 2025; Nakamura and Matsumoto, 2025). However, the immune environment typically varies with tissue changes due to the adaptation of tissues to different functional requirements. The oral cavity, adapted for functions such as mastication and serving as the first portal of the digestive tract, has fostered a mucosal environment that continuously undergoes physical and microbial stimuli while maintaining a high level of immunity. These differences are evident in the composition of immune cells, levels of inflammatory response, microbial interactions, healing mechanisms, and immune tolerance strategies. In oral mucosal diseases such as recurrent aphthous ulcers (RAU) (Wang Z. et al., 2025), oral candidiasis (Saraswat et al., 2025), and Behçet’s disease (BD) (Zhang et al., 2024), an increase in neutrophil infiltration and alterations in neutrophil function are commonly observed, indicating their involvement in the pathological process. Similarly, in oral cancer, neutrophils exhibit functional heterogeneity influenced by the microenvironment, thereby affecting the biological behavior of the tumor (Zheng et al., 2022). Although there is increasing recognition of the crucial role of neutrophils in specific oral mucosal diseases, the precise molecular mechanisms by which neutrophils contribute to the failure of immune defense, the initiation and progression of pathological damage remain incompletely understood within the unique oral mucosal environment. Consequently, we will investigate the specificity of the oral mucosal environment and its impact on neutrophils, beginning with an exploration of neutrophils’ functions and mechanisms, and systematically summarizing their alterations in oral mucosal diseases. By integrating the latest research advancements, this paper aims to provide new perspectives on understanding the mechanisms underlying oral mucosal diseases and to establish a theoretical foundation for related translational research.

## 2 Neutrophils and oral mucosa

Crucially, neutrophils are not merely transient responders to infection in the oral cavity; they are constitutively present and strategically positioned within the oral mucosal barrier. Even under healthy conditions, neutrophils actively patrol key sites like the junctional epithelium and are readily detectable in saliva, gingival crevicular fluid, and the dental biofilm (Schönherr et al., 2017). This baseline presence underscores their fundamental role as the sentinels and first line of defense of the oral mucosa, continuously surveilling and responding to the constant microbial and mechanical challenges inherent to the oral environment (Silva et al., 2023). Understanding how this critical population is maintained—through continuous production and precise recruitment—is essential to grasp their function in oral mucosal health and disease.

### 2.1 Role of neutrophils and its mechanism

#### 2.1.1 The production and recruitment of neutrophils

**Figure 1:**
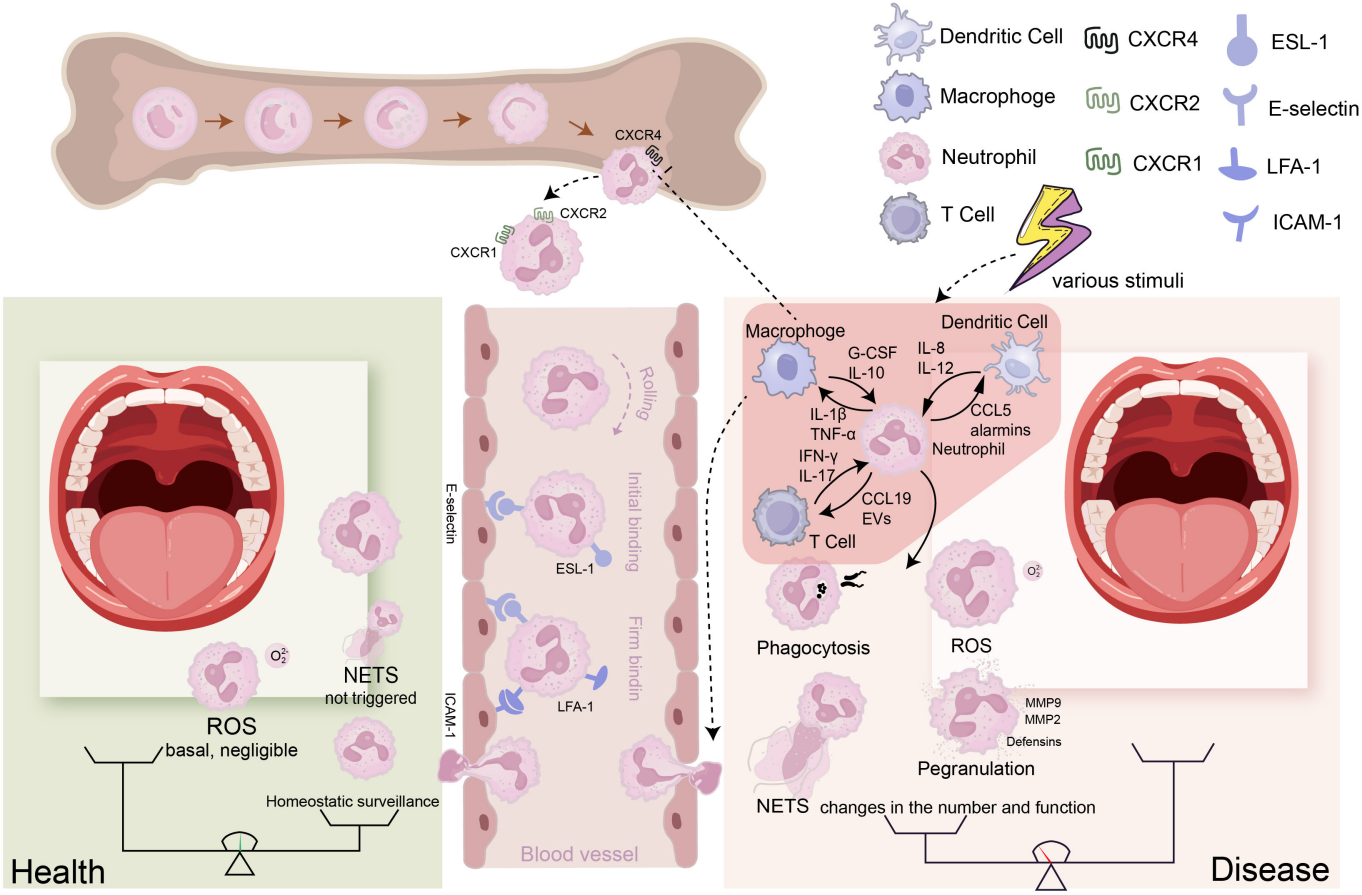
The transition of neutrophils from a healthy state to a disease state: Neutrophils are generated in the bone marrow, progressing through several stages: hematopoietic stem cells (HSCs), granulocyte-monocyte progenitors (GMPs), precursor neutrophils (PreNeu), and mature neutrophils. In the bone marrow, neutrophils express high levels of CXCR4; when this receptor is inhibited by G-SCF, neutrophils lose their affinity for the bone marrow and exit it. Neutrophils that leave the bone marrow express high levels of CXCR1/2. To reach the corresponding sites via the bloodstream, neutrophils must undergo rolling, capture, crawling, and migration processes to enter the tissues. During this process, E-selectin mediates initial adhesion, while ICAM-1 ensures firm binding. (Left) Under healthy conditions, only a small number of neutrophils patrol at low levels in the tissues, and ROS production or NET formation is minimal, maintaining tissue homeostasis. (Right) Under disease conditions, various stimuli lead to the production of cytokines by different cells, attracting a large number of neutrophils into the bloodstream and facilitating their entry into the affected tissues under the influence of chemokine gradients. The neutrophils entering the tissues can further interact with these cells, while their effector functions (such as NET formation, degranulation, ROS production, and phagocytosis) change, resulting in an imbalance between immunity and destruction that causes tissue pathology.

Neutrophils arise from hematopoietic stem cells (HSCs) in the bone marrow differentiating via granulocyte-monocyte progenitors (GMPs) through stages (myeloblasts to band cells) under G-CSF regulation to become mature neutrophils. Approximately 10^11^ PMNs are produced daily. Without inflammation, fewer than 12% of these cells enter the bloodstream (Kraus and Gruber, 2021). Stimulation downregulates CXC chemokine receptor (CXCR) -2 and upregulates CXCR4, mobilizing neutrophils from marrow into circulation (Eash et al., 2010; Casanova-Acebes et al., 2018). This process can be summarized as follows: under the influence of cytokines such as G-CSF, CXC chemokine ligand (CXCL)-1, CXCL2, C-C motif chemokine ligand (CCL)-3, interleukin (IL) -1β, and Intercellular Cell Adhesion Molecule (ICAM)-1, particularly G-CSF, which is the primary cytokine regulating neutrophil recruitment and development (Martin et al., 2021). Neutrophils continuously enter systemic circulation. At inflammation sites, they undergo extravasation: initial selectin (E-, L-selectin)-mediated rolling on endothelium, followed by integrin (LFA-1, Mac-1)-dependent firm adhesion via ICAM1 binding (Maier-Begandt et al., 2024). Guided by chemokines (e.g., CXCL1, CXCL2, IL-1α) from resident cells, neutrophils then transmigrate across the vascular wall via PECAM-1 and CD99-mediated pathways (paracellular or transcellular) (Xia et al., 2024). Within inflamed tissues, they migrate along chemotactic gradients to perform immune functions (**Figure 1**).

#### 2.1.2 The role of neutrophils and their crosstalk with other cells

Neutrophils immune functions involve phagocytosis, degranulation (primary, secondary, tertiary, secretory vesicles), ROS production (superoxide, H_2_O_2_, HOCl), and NET release (nuclear/mitochondrial DNA) (Riaz and Sohn, 2023). Their core sterilization function eliminates phagocytosed pathogens within phagosomes via granule components (e.g. neutrophil elastase (NE) and myeloperoxidase (MPO)) and ROS. Larger microbes like *C. albicans* are targeted via NETosis (Branzk et al., 2014). NETs comprise DNA (nuclear/mitochondrial), histones, granule antimicrobial proteins (Jeon et al., 2024). Additionally, neutrophils also exhibit atypical roles in wound healing, repair, and angiogenesis (Hsu et al., 2025; Vicanolo et al., 2025). Neutrophils are the initial non-resident cells that reach infected or inflamed tissues, providing them with a selective advantage in managing the migration and activation of additional immune cells. The neutrophil proteome, which is abundant in cytokines, chemokines, and antimicrobial peptides, plays a crucial role in intercellular communication and interact with various immune and non-immune cells. For example, neutrophil-derived chemokines (CCL3/4/5/20) and alarmins (α-defensins, anionic peptides, HMGB1) attract dendritic cells (Schuster et al., 2013). Pro-inflammatory factors such as IL-1β and Tumor Necrosis Factor-α (TNF-α) released by neutrophils can activate macrophages and enhance their phagocytic and antigen-presenting capabilities (Tsai et al., 2021). Other proteins released by neutrophils, such as Cathelicidin antimicrobial peptide LL-37 (LL-37), proteinase 3, uromodulin, and cathepsin G (Cat-G) (Soehnlein et al., 2009; Walters et al., 2021), can also activate the innate immune response.

In recent years, it has been confirmed that neutrophils can interact with lymphocytes through various mechanisms, particularly evident in the immune regulation processes of cancer and autoimmune diseases. In the tumor microenvironment, tumor-associated neutrophils (TANs) inhibit T cell activity by expressing PD-L1, thereby weakening T cell metabolic functions (Deng et al., 2021); the NETs they form can also physically hinder T cell infiltration and activate immunosuppressive signaling pathways, promoting immune evasion (Zuo et al., 2023). Conversely, under specific conditions, neutrophils can respond to signals such as IFN-γ, enhancing the anti-tumor responses of CD8+ T cells and NK cells, reflecting their functional plasticity (Sun et al., 2014). In autoimmune diseases, such interactions tend to drive pathological inflammation. In rheumatoid arthritis, neutrophil cytosolic factor 1 (NCF1), as a core subunit of NOX2, participates in neutrophil migration, maturation, and CXCR2 expression by regulating ROS production, and promotes Th1/Th17 differentiation, revealing the interaction mechanisms between neutrophils and T helper cells (Chen et al., 2024b). In systemic lupus erythematosus, NETs serve as carriers of autoantigens, activating dendritic cells and B cells, inducing the production of autoantibodies and exacerbating tissue damage (Chapman et al., 2019). In addition, these cells have the ability to positively or negatively regulate T cell and B cell subsets (Costa et al., 2019). For example, neutrophil-derived extracellular vesicles can regulate the activation and differentiation of T cells by delivering miRNA or protein molecules (Pfister, 2022).

Meanwhile, neutrophils are also influenced by other immune cells. Macrophages can regulate the activity of neutrophils by secreting anti-inflammatory factors such as IL-10, thereby achieving a fine balance in the inflammatory response (Awasthi and Sarode, 2024). M2 macrophage-derived extracellular vesicles can disrupt the maturation trajectory of neutrophils, with key reprogramming genes driving the differentiation of the repair subset Anxa1^hi^ (Yao et al., 2025). Under microbial stimulation, a unique population of epithelial cells and fibroblasts in the oral mucosa of healthy individuals, which express CXCL1, CXCL2, and CXCL8, can also maintain immune homeostasis by recruiting neutrophils (Williams et al., 2021). Depending on the tissue, pathogen, and interacting cells, the signaling pathways activated during the stages of neutrophil activation and functional roles may vary. These differing signaling pathways, located upstream and downstream of neutrophils, influence the role of neutrophils in tissues.

### 2.2 Oral environment and its impact on neutrophils

Tissue-specific cues are crucial for maintaining the homeostasis of the mucosal barrier. Unlike other tissues, the oral mucosa, even in a healthy state, allows neutrophils to traverse the epithelial connection between the teeth and the mucosa (the junctional epithelium) and reach the periodontal pocket (Uriarte and Hajishengallis, 2023). In the oral cavity, neutrophils have been detected in tissues, saliva, dental biofilm, and gingival crevicular fluid. This phenomenon may be related to the unique environment of the oral cavity.

#### 2.2.1 The impact of oral microbiota on neutrophils

The oral cavity represents the second largest site of microbial colonization in the human body, following the intestines. The traits and regulatory systems of the oral microbiome are crucial in maintaining both health and disease. This microbiome is composed of a diverse array of microorganisms, including bacteria, fungi, viruses, and protozoa, which collectively form a complex ecological network. These microorganisms colonize and interact within various oral microenvironments, such as the tooth surface, gingival sulcus, and tongue.

The oral environment harbors a high microbial load. In this section, we interpret the impact of microbes on neutrophils by presenting several recent reports on microbes that play key roles in oral microecology. These microbes have been widely reported in the two most common oral diseases, caries and periodontal disease, and recent studies indicate that their roles in oral mucosal diseases are gaining increasing attention. For instance, *Porphyromonas gingivalis* and *Fusobacterium nucleatum* are closely associated with OSCC. The abundance of *P. gingivalis* and *F. nucleatum* is significantly elevated in OSCC tissues (Federica et al., 2023), and they can promote tumor progression through signaling pathways such as IL-6/STAT3 and E-cadherin/β-catenin (Wang et al., 2024a). Some studies suggest that the abundance of *F. nucleatum* in the buccal mucosa of patients with OLP is significantly increased (Du et al., 2020). However, the pathogenic mechanisms of these microbes in other oral mucosal diseases require further investigation. Theoretically, their interaction with neutrophils and the host is an indispensable part of the pathogenic mechanism. Different microbes possess distinct structures, which finely regulate the functional activity of neutrophils through specific recognition of microbes and their products. Specifically, this regulatory process primarily relies on the recognition of pathogen-associated molecular patterns (PAMPs) and microbial metabolites by pattern recognition receptors (PRRs) expressed on the surface of neutrophils (Ji and Choi, 2013). In the complex oral microecology, different categories of microorganisms influence key functional outputs of neutrophils through downstream signaling pathways activated by different receptors. For instance, the lipopolysaccharide (LPS) from the Gram-negative bacterium *Porphyromonas gingivalis* relies on TLR4-mediated signaling, while its fimbriae activate pro-inflammatory pathways via TLR2, enhancing neutrophil reactivity (Nativel et al., 2017). In contrast, Gram-positive bacteria such as *S. mutans* and *Fusobacterium nucleatum* primarily expose lipoteichoic acid (LTA) and peptidoglycan (PGN) as PAMPs, which are recognized by TLR2. Some degradation products can also enter the cell and be sensed by the NOD2 receptor, collectively driving the amplification of inflammatory signaling (Eisenreich W, 2017). In addition to structural PAMPs, microbial metabolites and the matrix components of biofilms are also important signaling molecules. These metabolites are sensed by neutrophils in a manner that may or may not depend on PRRs. For instance, a mixture of short-chain fatty acids in the supernatant of *P. gingivalis* has been shown to specifically recruit neutrophils through the cell-selective free fatty acid receptor 2 (FFAR2), while other leukocytes, such as monocytes, do not respond to this stimulus (Dahlstrand et al, 2020). Meanwhile, extracellular DNA (eDNA) in biofilms is rich in unmethylated CpG motifs, which can be recognized by intracellular TLR9 (Almaarik et al., 2025). Following the recognition of microbes, the functions of neutrophils also undergo corresponding changes. For instance, after TLR activation, the MyD88 or TRIF-dependent pathways induce the NF-κB and MAPK pathways, promoting the release of inflammatory factors from neutrophils (Kawai and Akira, 2007). The activation of NOD1/NOD2 can induce the expression of antimicrobial peptides in neutrophils through the NF-κB pathway (Biber et al., 2025).

#### 2.2.2 The impact of oral function on neutrophils

The oral cavity, as the initial entry point of the digestive system, also functions in food decomposition. The saliva and mastication required during the process of food decomposition also influence neutrophils. Mechanical stimulation during mastication can affect the barrier function of the oral mucosa through various signaling pathways. For instance, The mechanical force of mastication stimulates gingival epithelial cells to secrete IL-6, driving the accumulation of Th17 cells, which in turn increases the expression of neutrophil chemotactic factors (such as IL-17), thereby enhancing the chemotaxis and recruitment of neutrophils (Dutzan et al., 2017). Fibrinogen in saliva, by binding to the integrin receptor αMβ2, activates the effector functions of neutrophils, including the production of ROS and the formation of NETs, thereby transforming neutrophil immunity from protective to destructive. This may serve as a pathogenic trigger for common mucosal diseases (Silva et al., 2021).

#### 2.2.3 The impact of systemic factors on neutrophils

The oral environment is characterized by fluctuations influenced by factors such as time, diet, smoking, and host genetics. Systemic factors, including diabetes and hypertension, exhibit a bidirectional relationship with oral health. It is well-established that the prevalence of periodontitis among diabetic patients is 2–3 times higher than that in non-diabetic individuals (Atkin and Cowie, 2024). A recent study has revealed the impact of a high-sugar environment on neutrophils, thereby impairing the oral mucosal immune barrier. Wang et al (Wang et al., 2024c)found that NETs were prominently present at the site of oral mucosal lesions in mice, and inhibiting NETs could alleviate inflammation and damage. Through experiments, it was demonstrated that hyperglycemia promotes the formation of NETs via glucose transporter 1-mediated glycolysis in neutrophils, leading to mucosal barrier disruption.

## 3 Neutrophil cells in human oral mucosal diseases

Oral mucosal diseases as a common category of diseases, have complex etiologies that remain not fully elucidated. The lack of targeted and effective treatment options in clinical practice not only affects the quality of life of patients but also poses ongoing challenges for medical research. For a long time, the immune-inflammatory response has garnered widespread attention in the development of oral mucosal diseases, particularly in typical conditions such as recurrent aphthous stomatitis, Behçet’s disease, oral potentially malignant disorders, and oral cancer, where abnormal activation of immune cells and imbalance of immune regulation are considered core driving factors of disease progression. Among various immune cells, neutrophils, as the first line of defense in the innate immune system, have increasingly highlighted their crucial role in local inflammation and immune responses within the oral mucosa. Therefore, we will systematically analyze the recruitment, activation, functional abnormalities of neutrophils, and their interactions with other immune cells in different oral mucosal diseases, providing a theoretical basis for revealing the immunological foundations of the occurrence and development of various oral mucosal diseases, as well as for identifying new therapeutic targets and developing targeted treatment strategies.

### 3.1 Neutrophil in oral candidiasis

Oral candidiasis is a prevalent oral infection caused by fungi of the *Candida genus*, representing an opportunistic infection that typically affects individuals with compromised immune function or altered oral microenvironments. It is characterized by fungal overgrowth, microbial dysbiosis, and host inflammatory responses. Neutrophils are key cells in the host inflammatory response, and understanding their behavior in this disease is crucial for deciphering the pathogenesis of candidiasis.

#### 3.1.1 Chemotaxis and activation of neutrophils by Candida

**Figure 2:**
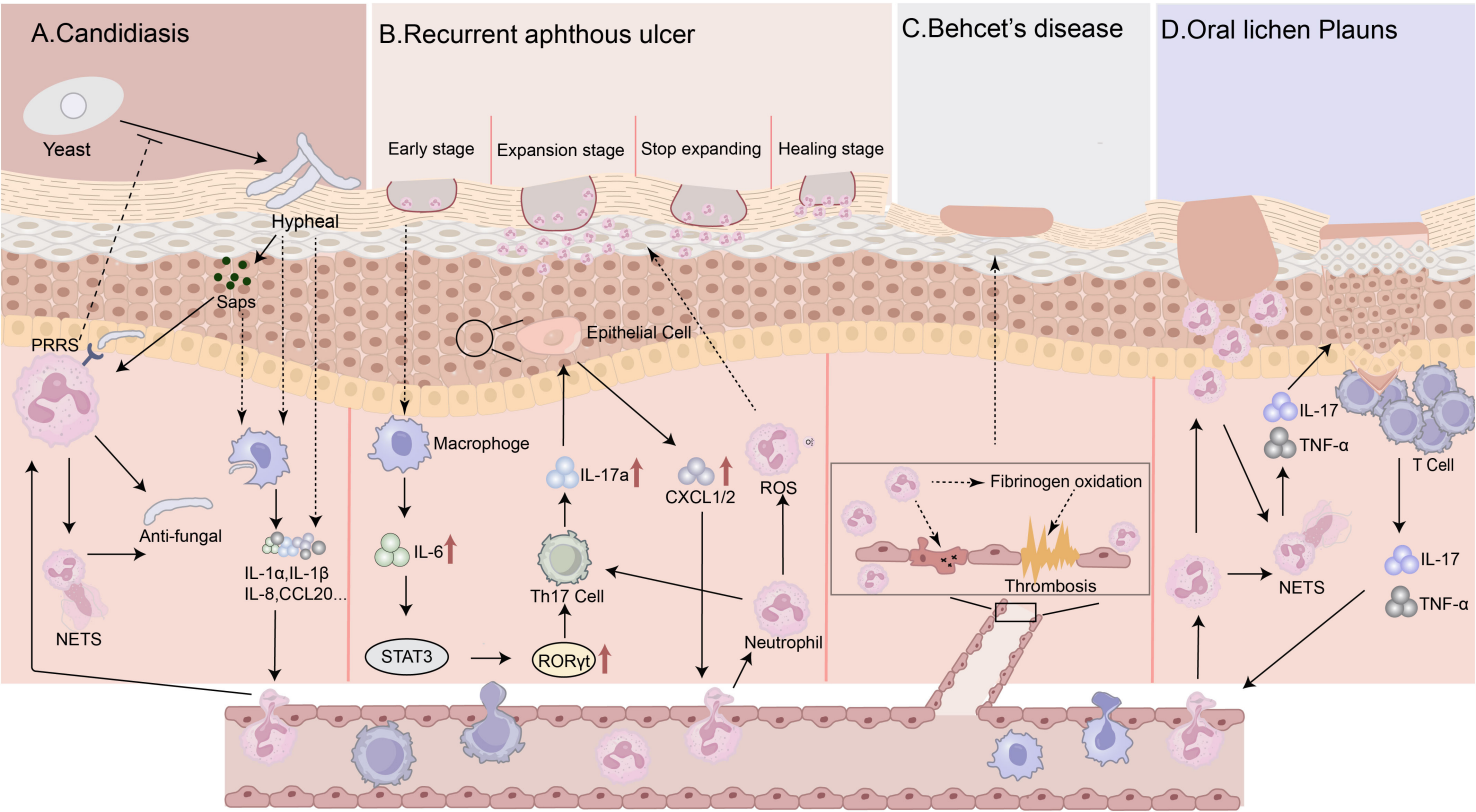
Mechanisms of neutrophils in oral mucosa disease: **(A)** In oral candidiasis, the hyphal form of Candida and its secreted virulence factors can be recognized by macrophages, which then secrete cytokines to recruit neutrophils to the affected area. Neutrophils that reach the tissue first can inhibit the transition of Candida from the yeast form to the hyphal form. Additionally, neutrophils can recognize and activate against Candida through two mechanisms: one involves several pattern recognition receptors (PRRs) on their surface, and the other is a direct response to virulence factors of Candida albicans, such as Saps. Activated neutrophils can exert antimicrobial effects through mechanisms such as the formation of neutrophil extracellular traps (NETs). **(B)** In recurrent aphthous ulcers, neutrophils are initially localized to the epithelial fissures and the fibrin clots on the surface of the ulcers during the early stages of ulcer formation. As the ulcer expands, neutrophils gradually infiltrate the adjacent and underlying tissues. Once the lesion enlargement stops, the number of neutrophils decreases in areas distant from the ulcer. During the healing phase, neutrophils dominate at the top of the lesion. In the initial stage, macrophage activation due to external stimuli leads to an increase in IL-6 secretion. IL-6 activates the STAT3 signaling pathway, resulting in increased expression of the transcription factor RORγt, which further amplifies Th17 cells. Th17 cells secrete IL-17a to promote epithelial cells to express CXCL1/CXCL2, thereby recruiting neutrophils. Neutrophils, in turn, enhance Th17 activity through unknown signals and further exacerbate mucosal barrier damage by releasing reactive oxygen species (ROS) and other factors. **(C)** In Behçet’s disease, there is significant neutrophil infiltration and abnormal activation, and the cytokines from activated neutrophils can oxidize prothrombin, promoting thrombosis and maintaining a hypercoagulable state; they can also directly or indirectly cause endothelial cell damage, further exacerbating vasculitis. **(D)** In oral lichen planus, the levels of inflammatory cytokines IL-17 and TNF-α are elevated at the lesion sites, which attracts neutrophils to the lesions and exacerbates the inflammatory response.

*Candida albicans*, the main pathogen in oral candidiasis, derives significant pathogenicity from its yeast-to-hyphal morphological transition. Neutrophils are crucial for defense against mucosal *Candida* infections, being uniquely capable of inhibiting this transition (Saraswat et al., 2025). *Candida* triggers local inflammation by releasing pro-inflammatory factors (e.g., G-CSF, CCL20, IL-1α, IL-1β, IL-8, β-defensin), activating host immunity. Neutrophils respond to these signals, migrating to infected tissue (Zhao et al., 2024). At the infection site, neutrophil PRRs – including Dectin-1/2/3, Toll-like receptor (TLR)2/4/9, macrophage-inducible C-type lectin (MINCLE) and DC-SIGN – recognize *C. albicans* antigens, leading to activation (Zhu et al., 2023). Neutrophils can also respond directly to fungal virulence factors, such as aspartyl proteases (Saps) (Zawrotniak et al., 2017) (**Figure 2**).

As the infection transitions from the free-floating bacterial phase to the more complex biofilm stage. On one hand, the excessive or sustained activation of neutrophils, although intended to eliminate pathogens, also leads to collateral damage to host tissues due to the release of large amounts of proteases, reactive oxygen species, and other substances (Metzemaekers et al., 2020). On the other hand, certain responses from neutrophils may inadvertently allow the progression of the infection and support the survival of microorganisms within the biofilm. Firstly, mature *C. albicans* biofilms can actively inhibit the release of extracellular traps by neutrophils through their extracellular matrix, thereby weakening this critical defense weapon and significantly enhancing the immune evasion and persistence of the yeast (Johnson et al., 2016). Secondly, although NETs have a bactericidal effect in clearing free-floating *C. albicans*, their efficacy is limited in the context of biofilms. Consequently, under the dual impact of inflammation and tissue damage, local increases in nutrients and adhesion sites may occur on the oral surface, thereby promoting the consolidation and reconstruction of biofilms (Kernien et al., 2020). The fungi themselves can also evade neutrophil clearance through various mechanisms. For instance, *Staphylococcus aureus* can encapsulate *C. albicans*, thus collaboratively evading neutrophil extracellular traps, and in the presence of neutrophils, *Staphylococcus aureus* enhances the proliferation and hyphal growth of *C. albicans* (Jing et al., 2024). It is noteworthy that the extracellular matrix of biofilms is rich in extracellular DNA (eDNA), which is crucial for maintaining structural integrity and drug resistance (Rajendran et al., 2014). Given that the framework of NETs consists of decondensed chromatin DNA, it is theoretically possible that the DNA framework released by neutrophils in NETs could be integrated into the matrix of biofilms, thereby enhancing their physical stability (Whitchurch et al., 2002). However, there is currently a lack of direct and reproducible experimental evidence to confirm the hypothesis that “NETs act as scaffolds for *C. albicans* biofilms” in oral mucosal models.

#### 3.1.2 Neutrophil-mediated killing of Candida and its mechanisms

Activated neutrophils combat *C. albicans* via direct killing and immune collaboration. Direct killing occurs through three primary mechanisms: The first is Extracellular Killing - targeting unphagocytosed fungi via complement receptor 3 and caspase recruitment domain-containing protein 9, and, releasing MPO, defensins, and lactoferrin (Feldman et al., 2019). The second is Phagocytic Killing - eliminating internalized fungi via Fcγ receptors, protein kinase C (PKC), and NADPH oxidase-dependent pathways, utilizing oxidative (ROS) and non-oxidative mechanisms (MPO, defensins, lactoferrin) (Feldman et al., 2019). ROS generation involves NADPH oxidase producing O_2_^-^, converted to potent oxidants like HOCl, causing DNA damage and protein oxidation (Ulfig and Leichert, 2021). The third is NETosis - releasing NETs stimulated by *C. albicans* hyphae. NETs physically trap and enzymatically degrade hyphae, crucial as their size impedes phagocytosis (He et al., 2022).

In terms of synergistic effects, Th17 cells not only influence the recruitment of neutrophils via IL-17 but also promote epithelial cells to secrete antimicrobial peptides such as β-defensin-3, thereby enhancing the bactericidal activity of neutrophils (Conti et al., 2009). Salivary IgA inhibits Candida adhesion through agglutination, while lactoferrin and lysozyme directly disrupt the fungal cell wall, creating favorable conditions for neutrophil phagocytosis. Reduced saliva flow (e.g. xerostomia) weakens these auxiliary effects and increases the risk of infection (Pedersen et al., 2018). Additionally, IL-22 released by neutrophils enhances the expression of epithelial tight junction proteins, which repair barrier function and prevent fungal dissemination (Beute et al., 2022).

### 3.2 Neutrophil in recurrent aphthous ulcer

Recurrent aphthous ulcer (RAU), is the most prevalent type of oral mucosal ulcerative disease. Histopathological findings indicate that aphthous ulcers in RAU patients are characterized by cell-mediated inflammation, primarily involving the infiltration of neutrophils, monocytes, and T cells (Mills et al., 1980). This suggests the crucial role of neutrophils in the pathological changes of RAU.

#### 3.2.1 Neutrophil infiltration dynamics in RAU

In the process of ulcer formation, neutrophils are initially confined to the epithelial breaches and fibrin clots on the ulcer surface, subsequently spreading to adjacent and underlying tissues. As the expansion of the lesion ceases, the number of neutrophils in the tissues adjacent to, but distant from, the ulcer decreases. In the healing phase, neutrophils predominantly occupy the lesion’s top (Stanley, 1972; Mills et al., 1980). The neutrophil-to-lymphocyte ratio (NLR), an inflammation/stress marker, is also studied in RAU, though findings are conflicting. Several studies (Terzi et al., 2016; Kayabasi et al., 2019; Uluyol and Kilicaslan, 2019; Atalay F, 2022) and one meta-analysis (Rahimi et al., 2024) report elevated NLR in RAU patients, while others (Karaer, 2020; Turan et al., 2021) found no statistically significant difference compared to controls. Collectively, neutrophils appear associated with RAU. Collectively, the literature suggests that neutrophils are associated with RAU to some extent.

Furthermore, Neutrophil function in RAU has also been investigated. Reports on phagocytic function are contradictory: Lukac (Lukac et al., 2003) and Kumar (Kumar et al., 2010) described reduced phagocytosis in saliva (and peripheral blood (Kumar et al., 2010)) of RAU patients versus controls, whereas Altinor (Altinor et al., 2003) suggested enhanced function. Chemotaxis findings are inconsistent: Sistig (Sistig et al., 2001) observed significantly reduced blood neutrophil chemotaxis in RAU patients, partially recovering post-remission; Dagalis, however, found no difference in chemotaxis or spontaneous migration versus controls. Regarding oxidative stress, an imbalance (excess free radicals, insufficient antioxidants) may directly damage oral epithelial cells by activating neutrophils to release proteases and ROS (Dagalis et al., 1987). A recent systematic review (Estornut et al., 2024) synthesized evidence showing elevated oxidative stress in RAU, marked by significantly increased oxidative markers and decreased antioxidants in both saliva and blood.

#### 3.2.2 Possible pathogenic and regenerative mechanisms of neutrophils in the RAU

In terms of mechanisms, the latest study found a significant increase in neutrophil infiltration in the tongue mucosal tissue of a chemical stomatitis (CS) mouse model, accompanied by a higher percentage of Th17 and Th1 cells. After directly targeting and depleting neutrophils with anti-Ly6G antibody, there was a notable reduction in the local inflammatory response in CS mice, along with a simultaneous decrease in the infiltration of Th17 and Th1 cells. This confirmed the presence of a positive feedback regulatory loop between Th17 cells and neutrophils in stomatitis and revealed the critical role of the IL-6-Th17-neutrophil axis in the pathogenesis of stomatitis (Wang Z. et al., 2025) (**Figure 2**). However, the mechanisms surrounding neutrophils in recurrent aphthous ulcer remain in the emerging stage. Recent research on ulcer-related diseases has provided significant insights into understanding the pathophysiological mechanisms of RAU and identifying new therapeutic targets. For instance, In diabetic foot ulcers, the NET release of neutrophils in diabetic wound tissue is regulated by the NLRP3/caspase-1/GSDMD pathway, and the use of disulfiram to inhibit GSDMD can suppress this pathway, reducing NET formation and thus accelerating diabetic wound healing (Yang et al., 2023). Notably, there is a marked infiltration of neutrophils in the lesion areas of RAU. However, whether GSDMD-mediated NETosis is involved in the process of oral epithelial damage and ulcer formation remains a critical scientific question that urgently needs to be elucidated.

In terms of healing mechanisms, studies using animal models support the notion that the depletion of neutrophils accelerates the epithelial regeneration of wounds (Dovi et al., 2003), while also demonstrating that the absence of neutrophils can delay wound healing (Leppkes et al., 2022). This suggests the complexity of neutrophil functions, as a simple characterization of ‘promotion’ or ‘inhibition’ may not encompass their entire role. Previous discussions have shown that neutrophils infiltrate various stages of RAU, especially tending to occupy the top of lesions during the healing phase. In fact, in the study of ulcer-associated diseases, the ‘regenerative function’ of neutrophils is not in stark opposition to their ‘pathogenic role’. Rather, it represents a functional reorganization of the same cell population within the context of spatiotemporal and metabolic reprogramming. For instance, effective clearance of apoptotic neutrophils can drive the release of pro-repair factors and facilitate inflammation ‘programmed resolution’ (Frangogiannis, 2012). Recent studies have also found that neutrophils are involved in the construction of extracellular matrix, forming a ‘wall’ that defends against foreign molecules and bacteria (Vicanolo et al., 2025). Neutrophil extracellular traps (NETs) induce fibroblast ferroptosis through endoplasmic reticulum stress, contributing to impaired wound healing in diabetes (Zhao and Liu, 2025). Future research could clarify the characteristic markers and regulatory axes of neutrophils as ‘pro-inflammatory’ versus ‘pro-regenerative’, and validate precise intervention strategies targeting timing and functional states in animal models and clinical studies, potentially transforming neutrophils from ‘pathological amplifiers’ into ‘regenerative facilitators’.

### 3.3 Neutrophil in Behçet’s disease

Behçet’s disease (BD) is a chronic, multisystem inflammatory vasculitis of unknown etiology, characterized by oral ulcers, genital ulcers, skin lesions and uveitis. Neutrophil hyperfunction has been implicated in BD pathogenesis since 1975 (Matsumura and Mizushima, 1975). Interpreting the changes in neutrophils in BD serves as a crucial foundation for studying the pathogenesis and therapeutic approaches of BD.

#### 3.3.1 Neutrophil activation and dysfunction in BD

The Pathological hallmarks of BD include excessive neutrophil activation and tissue infiltration, driving manifestations from oral ulcers to CNS damage (Neves and Spiller, 2013; Cho et al., 2014). Recent multi-omics analysis identified persistent upregulation of neutrophil chemotaxis mediators (CXCL8, TLR4) across systemic, immune, and ocular tissues in BD, suggesting enhanced chemotaxis as a disease hallmark. Disease progression involves enhanced chemotaxis, endothelial injury, hemostasis activation, and oxidative stress (Pu et al., 2025). Transcriptomics confirm enrichment of innate immunity, signaling, and chemotaxis pathways. Increased neutrophil infiltration co-localizes with PDE4 in BD skin lesions (Le Joncour et al., 2023).

Nearly all neutrophil functions are altered in BD patients, though reports conflict. This may indicate disease arises from either excessive activation or impairment. Some propose pre-activation *in vivo* leads to reduce *in vitro* bactericidal activity and “exhaustion,” blunting chemotactic responses, especially in severe disease. Chemotaxis studies conflict: some report active BD serum stimulates E-selectin, ICAM-1, and CD11α/CD18 expression on healthy neutrophils (Sahin et al., 1996; Triolo et al., 1999), while others found no adhesion difference (Carletto et al., 1997). Phagocytic activity is reported as lower (Murad et al., 2022) or unchanged (Eksioglu-Demiralp et al., 2001; Atalay et al., 2002; Perazzio et al., 2015). ROS generation findings diverge: reduced (Eksioglu-Demiralp et al., 2001; Murad et al., 2022) vs. increased (FMLP-stimulated) (Takeno et al., 1995). Degranulation studies show elevated NE, α-defensin 1-3, metalloproteinase-9(MMP9), and MPO activities (Değer et al., 1995; Köse et al., 1995; Accardo-Palumbo et al., 2000; Mumcu et al., 2012; Shaat et al., 2020), though MMP9 levels were unchanged in one study (Aksoy et al., 2011). Regarding NETosis, BD neutrophils exhibit increased spontaneous NETosis (Perazzio et al., 2017). Active BD plasma more effectively stimulates NET release than healthy or remission plasma (Safi et al., 2018). NETosis markers (MPO-DNA complexes (Le Joncour et al., 2019), dsDNA (Li et al., 2021)) are significantly elevated in BD patients.

#### 3.3.2 Pathogenic mechanisms of neutrophils in autoimmune diseases, particularly BD

Neutrophils have roles in autoimmune diseases that extend far beyond their traditional functions of phagocytosis and pathogen killing. They are increasingly recognized as central drivers of disease onset and progression. In the pathological processes of autoimmune diseases such as BD, the scaffolding of NETs is adorned with a plethora of highly immunogenic proteins. In an autoimmune environment, these proteins can serve as significant autoantigens that activate adaptive immune responses, thereby making NETs one of the core pathogenic mechanisms in autoimmune diseases. On one hand, NETs expose components such as DNA and histones, which are typically sequestered within the nucleus, to the immune system, thus breaking the immune tolerance of the host. This exposure of previously hidden antigens provides direct conditions for the activation of autoreactive T and B cells. On the other hand, NETs are rich in various enzymes, such as peptidylarginine deiminase 4 (PAD4), which catalyzes the conversion of arginine residues on proteins like histones to citrulline residues (i.e., citrullination) (Li et al., 2010), thereby forming new antigenic epitopes (neoantigens). These citrullinated proteins are considered important triggers for the production of anti-citrullinated peptide (CCP) antibodies in rheumatoid arthritis (RA) (Cooles et al., 2025). Similarly, components within NETs, such as myeloperoxidase (MPO) and proteinase 3, are also key target antigens in anti-neutrophil cytoplasmic antibody (ANCA)-associated vasculitis (Ray, 2012). The excessive formation of NETs and the obstacles to their clearance provide continuous antigen stimulation for autoreactive B cells and T cells, which is considered a key link in breaking self-tolerance, initiating, and maintaining autoimmune responses.

Specifically in BD, neutrophil alterations contribute to BD pathology, particularly via immunothrombosis. NETs enhance intravascular coagulation: thrombin generation rate/peak positively correlate with serum NET markers and decrease with DNase treatment. NETs activate both coagulation pathways and trigger thrombosis(Le Joncour et al., 2019). Furthermore, NETs induce endothelial dysfunction (reduced proliferation, increased apoptosis), enhance endothelial activation, and activate platelets (Safi et al., 2018). However, despite the established significant role of NETs in BD, our understanding of their precise components remains relatively limited. While NETs typically consist of common components such as DNA, histones, MPO, and NE, systematic studies focusing on the specific protein profiles and post-translational modifications (such as citrullination) of NETs in the circulatory systems of patients with BD, particularly those utilizing high-throughput techniques like mass spectrometry or quantitative proteomics, have yet to be comprehensively reported. This knowledge gap is critical, as NETs in different disease contexts may exhibit unique protein ‘fingerprints,’ and these specific components may be closely related to particular clinical phenotypes of BD (such as ocular, neurological, or gastrointestinal involvement). For instance, several differentially expressed proteins have been identified in the NETs of SLE and RA (Chapman et al., 2019), suggesting that a detailed molecular composition analysis of Behçet’s disease-specific NETs could provide important clues for discovering new disease biomarkers and potential therapeutic targets. Beyond NETs, neutrophils secrete pro-inflammatory cytokines (e.g. IL-1, TNF-α, IL-8), impacting endothelial cells and platelets (Zhang et al., 2023). Neutrophil activation also increases ROS production. ROS oxidize fibrinogen, impairing its function, altering fibrin polymerization, increasing plasmin susceptibility, elevating thrombogenicity, and enhancing clot stability – explaining BD’s pro-coagulant state (Becatti et al., 2016). Metabolites also affect neutrophils in BD. Elevated FPP excessively activates neutrophils via the calcium-TRPM2 pathway, exacerbating vascular inflammation and damage. Serum TNF upregulates TRPM2 expression, increasing neutrophil sensitivity to FPP. This creates a hyper-responsive state, leading to elevated serum NETs and cytokines (including TNF), establishing a pro-inflammatory positive feedback loop central to BD pathology (Zhang et al., 2024) (**Figure 2**).

### 3.4 Neutrophil in oral potentially malignant disorders

Oral Potentially Malignant Disorders (OPMDs) refer to a category of oral mucosal lesions with the potential risk of malignant transformation. This process results from the interaction of various local and systemic factors present in the oral cavity. Among these factors, the role of inflammation has garnered significant attention, as it alters the oral ecosystem, including the microbiome and immune components, leading to the formation of OPMDs and malignant lesions (Lenka et al., 2023). Neutrophils, as crucial participants in inflammation, have been reported in both OPMDs and oral cancer.

In oral lichen planus (OLP), the study by Cheng et al. suggests that neutrophils drive chronic inflammation and epithelial damage through the release of NETs and that IL-17 and TNF-α may mediate the formation of NETs in OLP patients (Cheng et al., 2024). In our previous studies, it has been demonstrated that MAIT cells producing IL-17A are increased in OLP. Therefore, the increased IL-17A-producing MAIT cells may interact with neutrophils in the inflammatory milieu of OLP (Chen et al., 2024a), which warrants further exploration (**Figure 2**).

In the context of oral leukoplakia (OLK), recent research has also focused on the role of neutrophils. TGF-β upregulates the transcription factors Bhlhe40 and Mxi1, which in turn promote the expression of RAGE and matrix metalloproteinase MMP9 in neutrophils. MMP9 cleaves membrane-bound RAGE to generate soluble RAGE (sRAGE), and sRAGE significantly promotes the malignant transformation of OLK through paracrine action (Chen et al., 2024c). However, the specific roles and mechanisms of neutrophils in the pathogenesis of both OLP and OLK remain incompletely understood. Future research should prioritize elucidating the precise molecular pathways governing neutrophil activation, NETosis, and their crosstalk with other immune cells in OLP, as well as validating the therapeutic potential of targeting neutrophil-derived mediators (such as sRAGE/MMP9 axis) in preventing OLK malignant progression.

### 3.5 Neutrophil in oral cancer

Oral squamous cell carcinoma (OSCC) represents a major type of oral cancer. Within the tumor microenvironment of OSCC and other cancers, neutrophils are referred to as tumor-associated neutrophils (TANs), exhibiting significant heterogeneity.

#### 3.5.1 Heterogeneity of neutrophils in oral cancer

**Figure 3:**
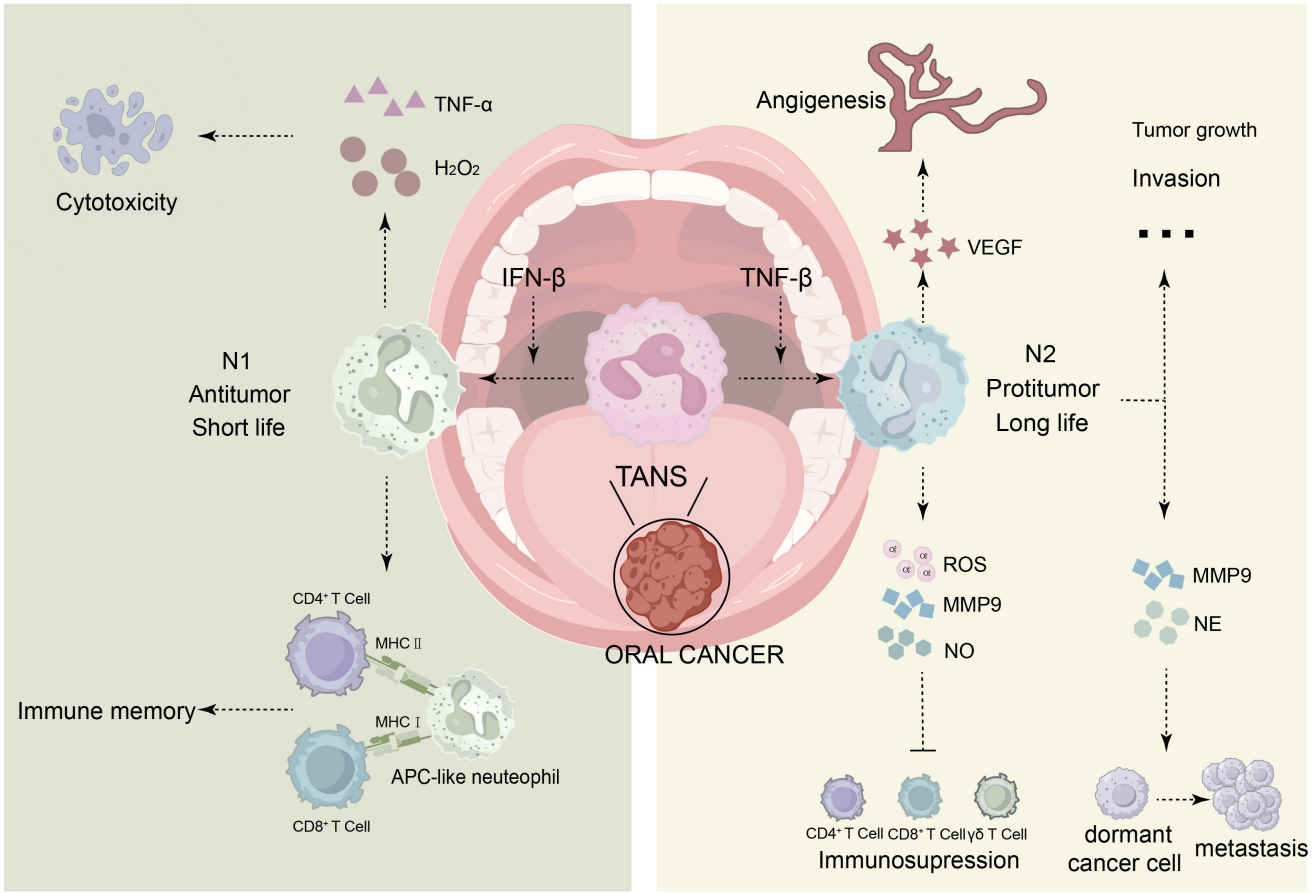
Heterogeneity of tumor-associated neutrophils (TANs) in the tumor microenvironment: TANs can be classified into two categories: N1 and N2. Typically, IFN-β mediates the differentiation of TANs into N1, while TNF-β mediates their differentiation into N2. N1 neutrophils have a shorter lifespan and exhibit anti-tumor properties; they can secrete TNF-α, H2O2, and other substances that exert cytotoxic effects and mediate tumor cell death. Additionally, they can present antigen information to facilitate immune memory. In contrast, N2 neutrophils have a longer lifespan and display pro-tumor characteristics. They secrete MPO, NE, ROS, MMP9, VEGF, among others, which can promote tumor growth, invasion, metastasis, angiogenesis, and immune suppression.

In cancer, Fridlender et al. pioneered the N1 (anti-tumor) vs. N2 (pro-tumor) classification (Fridlender et al., 2009). However, there is a viewpoint that strictly classifying TAN into N1 and N2 phenotypes oversimplifies their complexity (Zhou et al., 2025). To accurately delineate neutrophils in cancer, researchers have proposed a new cell type, polymorphonuclear myeloid-derived suppressor cells (PMN-MDSCs), which are immunosuppressive neutrophil-like cells that are abundantly present in cancer patients. Unlike N2-type TANs, PMN-MDSCs specifically refer to a highly specialized subpopulation of neutrophils that are induced under specific pathological conditions such as cancer, with their most defining feature being a potent immunosuppressive function (Guo et al., 2025). They not only directly suppress anti-tumor immune effector cells through mechanisms such as the production of reactive oxygen species (ROS) (Sprouse et al., 2019) and arginine consumption (Zhang et al., 2021), but also indirectly exacerbate immunosuppression by inducing regulatory T cells (Tregs) and remodeling the microenvironment (Tumino et al., 2021). In contrast, “N2” is a broader concept that encompasses a range of pro-tumor phenotypes associated with tissue repair, angiogenesis, and other processes. However, there is currently no consensus in the academic community on whether PMN-MDSCs represent a distinct type of neutrophil or merely different states of the same class of cells (Antuamwine et al., 2023).Recent single-cell transcriptome analysis across 17 cancers revealed neutrophils encompass at least 10 distinct states (e.g., inflammation, angiogenesis, antigen presentation) (Wu et al., 2024). Despite this diversity, the N1/N2 paradigm persists due to technical challenges in studying fragile, transcriptionally limited neutrophils. N1 TANs directly kill tumor cells via cytotoxic agents (H_2_O_2_, TNF-α), whereas N2 TANs promote tumor progression by secreting ROS, VEGF, and MMP-9 to induce angiogenesis, matrix degradation, and immunosuppression (Zheng et al., 2022). Theoretically, there is an intermediate state known as the N0 state, which has a neutral impact on tumors. Despite the functional differences between TANs, specific surface markers that can differentiate N1 from N2 still need to be identified, while the condition of neutrophils is mainly deduced from the expression of associated genes and molecules. Recently, a study integrated single-cell RNA sequencing data of Head and Neck Squamous Cell Carcinoma (HNSCC) and discovered significant heterogeneity among TANs, constructing a prognostic model based on characteristic genes of TANs (NRS). Among these, the neutrophil OLR1 gene was identified as a key biomarker that can influence patient survival by promoting tumor proliferation and migration (Cui et al., 2025). Circulating PMNs (cPMNs) comprise three density-based subsets: immature low-density neutrophils (LDNs: low CD16/CD11b, high CXCR2), mature LDNs, and mature high-density neutrophils (HDNs). HDNs resemble anti-tumor N1, while LDNs are dysfunctional and immunosuppressive. HDNs can convert to LDNs within the tumor microenvironment (TME) (Brandau et al., 2011; Hedric and Malanchi, 2022). Both TANs and cPMNs display phenotypic/functional plasticity influenced by the specific tumor context. Key polarizing factors are IFN-β (promoting N1) and TGF-β (promoting N2) (Jablonska et al., 2010, Jablonska et al., 2014) (**Figure 3**).

#### 3.5.2 The pathogenic mechanisms and significance of neutrophils in oral cancer

Numerous studies have extensively identified various mechanisms through which tumors interact with neutrophils, in cancer. For instance, N2-type TANs fuel tumor growth via ROS, which activates pro-survival pathways (NF-κB, PI3K/Akt/mTOR) in cancer cells, promotes angiogenesis, suppresses anti-tumor immunity (T cells, NK cells), and can cause DNA damage (Arfin et al., 2021; Sionov, 2021). Conversely, tumors manipulate neutrophil ROS production. For instance, tumor-secreted cathepsin C (CTSC) activates neutrophil protease 3 (PR3), triggering the CTSC-PR3-IL-1β axis. This boosts IL-6/CCL3 (recruiting more neutrophils), induces ROS/NETs, and ultimately supports metastasis (e.g., in lung cancer by degrading PAF-1) (Xiao et al., 2021). Similar interplay occurs in OSCC: VAP-1 downregulation hinders tumor growth by reducing NF-κB/IL-8 signaling and neutrophil infiltration (Xu et al., 2022), while Chemerin activates the JAK2/STAT3 pathway in OSCC cells via TANs, upregulating phosphorylated retinoblastoma, E2F transcription factor 1, cyclin E1/D1 (crucial cell cycle protein) to drive proliferation and metastasis (Hu et al., 2022). The net effect of these molecular interactions is an enhancement of tumor cell proliferation and metastasis, highlighting the significant role of Chemerin in the progression of OSCC. It is noteworthy that both N1 and N2 type TANs can produce ROS. However, the ROS produced by the former, such as H_2_O_2_, have a tendency to kill tumor cells and can exert anti-tumor effects when stimulated by signals such as TRAIL (Sionov, 2021).

In addition to ROS, TANs can also influence tumor cells through various mechanisms, with recent attention focusing on their regulation of the adaptive immune system. Data from pancreatic ductal adenocarcinoma (PDAC) indicate that a subset of TANs is negatively correlated with the infiltration of cytotoxic CD8^+^T cells in PDAC. This subset of TANs secretes a large amount of CCL5 and upregulates the membrane expression of Nectin2, promoting T cell dysfunction and enhancing the migration and invasion of cancer cells (Luo et al., 2024). The role of TANs in suppressing the adaptive immune system through NETs has also recently garnered attention. NETs, which have been discovered in multiple types of human cancers, have been reported to be intricately associated with tumorigenesis, metastasis, and tumor-related comorbidities. They can enhance tumor cell proliferation and promote metastasis through different processes, such as encouraging epithelial-mesenchymal transition, creating pre-metastatic niches, seizing circulating tumor cells and reactivating dormant tumor cells (Wang Y. et al., 2025). The tumor-promoting effects of NETs may be related to their inhibitory effects on T cells. Kaltenmeier et al. found in *in vitro* experiments that NETs utilize the immunosuppressive ligand PD-L1 to exhaust T cells, leading to their dysfunction (Kaltenmeier et al., 2021). Song et al. also confirmed that NET DNA binds to the transmembrane and coiled-coil domain 6 (TMCO6) on CD8^+^ T cells, impairing anti-tumor immunity and thereby promoting cancer progression (Song et al., 2024). In oral cancer, the high enrichment of NETs is associated with poor prognosis in OSCC. The NE in NETs inhibits pyroptosis in OSCC cells through NLRP3 mediation, thereby promoting their invasion and metastasis (Zhai et al., 2024). Overall, the paradoxical function of neutrophils in cancer, whether they support tumor growth or inhibit it, can be ascribed to the effects of the tumor microenvironment on their maturation, activation, and functional status. Currently, the NLR is considered an important prognostic indicator for OSCC in oral cancer (Magdum et al., 2024), but the translational research on neutrophils still requires further in-depth exploration.

### 3.6 Prospects of targeting neutrophils for the treatment of OMDs

In summary, neutrophils play a significant role in the pathogenesis of a range of oral mucosal diseases, from autoimmune inflammation to infections and malignancies, making targeted neutrophil therapy for oral mucosal diseases a viable option. By modulating neutrophil recruitment and effector functions, the pathological processes of these diseases can be altered.

Currently, mainstream targeting strategies include blocking the binding of chemokines to their receptors to prevent neutrophils from migrating from the bloodstream to sites of inflammation or tumors, with CXCR1/2 antagonists serving as representatives of this strategy (Komolafe and Pacurari, 2022). Additionally, intervening in key effector molecules released by neutrophils, such as NE, can alleviate tissue damage and the inflammatory cascade triggered by these molecules (Nishibata et al., 2024). NETs contribute to tissue damage in autoimmune diseases by promoting the production of autoantibodies and sustaining the inflammatory response (Wang et al., 2024b). Therefore, targeting the formation and clearance mechanisms of NETs has become one of the important strategies for treating autoimmune diseases of the oral mucosa. Regulating the generation and clearance of NETs by using DNase to degrade the DNA scaffold of NETs (Jordão et al., 2023), or by inhibiting key enzymes involved in the NETosis process (Le Joncour et al., 2023), can reduce the pathological damage caused by neutrophils. Finally, granulocyte colony-stimulating factor and its analogs have also been used to treat pathological damage caused by neutrophil deficiency (Campbell et al., 2016).

In the field of infectious oral diseases such as oral candidiasis, recent studies have reported that the application of DNase I enzyme combined with antimicrobial photodynamic therapy can significantly enhance the efficacy of photodynamic treatment (Le Joncour et al., 2023). In inflammatory and autoimmune diseases such as Behçet’s disease and recurrent aphthous ulcers, one of the optional strategies is to impede the migration of neutrophils to the inflammatory sites. In fact, the use of CXCR1/2 antagonists (such as Repertaxin) has demonstrated significant efficacy in reducing neutrophil influx and improving tissue damage in diseases like periodontitis (Huang et al., 2024b). In oral potentially malignant disorders (OPMDs) and oral cancer, neutrophils and their derivatives can not only serve as potential drug delivery vehicles (Naumenko et al., 2020) but can also be activated through specific inflammatory cascades to become powerful anti-tumor effector cells, providing new insights for cancer immunotherapy (Linde et al., 2023).

In addition to the pharmacological strategies targeting the recruitment and functional mechanisms of neutrophils, it is a question worth exploring whether lifestyle modifications that improve the oral environment can directly enhance neutrophil function, thereby providing a fundamental therapeutic strategy for oral mucosal diseases. As previously mentioned, the functions of neutrophils are finely regulated by various intrinsic and extrinsic factors. Emerging evidence suggests that certain modifiable lifestyle factors or adjustable systemic conditions, including overall metabolic status, dietary nutrition, physical exercise, psychological stress, and sleep rhythms, have been shown to significantly regulate neutrophils in both healthy individuals and disease models. Systemic metabolic conditions such as hyperglycemia can promote NETosis and impair the integrity of the mucosal barrier. Controlling blood glucose levels through regular medication can help manage the inflammatory state caused by neutrophils (Wang et al., 2024c). In terms of dietary nutrition, specific nutrients (such as n-3 polyunsaturated fatty acids and vitamin D) have shown anti-inflammatory and immunomodulatory properties. Studies indicate that these components can regulate the activation state of neutrophils, reduce their ability to produce inflammatory factors (such as IL-1β and TNF-α), and may promote the resolution of inflammation (Juan et al., 2019; Radzikowska et al., 2019). Regarding physical exercise, research indicates that regular exercise can improve innate immunity; moderate-intensity exercise performed regularly helps enhance the chemotaxis and phagocytic activity of neutrophils, and this effect can be sustained for a longer duration. Furthermore, chronic stress (Huang et al., 2024a) and sleep deprivation (Sang et al., 2023) can disrupt immune homeostasis. Conversely, interventions aimed at stress reduction and ensuring adequate high-quality sleep can help restore normal glucocorticoid rhythms and immune function (Yu et al., 2025), which may positively modulate neutrophil-mediated inflammation. Although improving lifestyle and systemic conditions alone may not reverse disease-specific neutrophil functional abnormalities or significantly improve disease progression, adopting lifestyle improvements and enhancing overall health as an adjunctive or preventive measure represents a promising non-pharmacological approach.

## 4 Discussion

As research progresses, there is a growing recognition of the diverse capabilities of neutrophils beyond their primary function of pathogen elimination. Neutrophils are also involved in transmitting signals for activation, inhibition, and migration; guiding antigen presentation; and regulating local immune responses. The literature reveals a rich repertoire of neutrophil behaviors, including the formation of neutrophil extracellular traps, reverse transendothelial migration, metabolic reprogramming, neutrophil aging, efferocytosis, and interactions with other cells. However, research on neutrophils in the context of oral mucosal diseases remains relatively limited, primarily focusing on their quantity and changes, with few studies investigating the mechanisms of neutrophil function within the unique oral cavity environment. Current literature indicates that the role of neutrophils in oral mucosal diseases is significant.

The current literature on how neutrophils precisely regulate disease progression and their relationship with the severity of clinical phenotypes exhibits significant heterogeneity and inconsistency. We believe this phenomenon is not coincidental but is instead rooted in the interplay of multidimensional research biases and biological complexities. Firstly, methodological differences across studies directly affect the comparability of results. For instance, in recurrent aphthous ulcers and Behçet’s disease, the severity of disease-related pain is often assessed using visual analog scales, which are subjective and may lead to varying correlations between neutrophils and clinical severity. Secondly, the diversity of study populations cannot be overlooked, including differences in ethnic backgrounds, lifestyle habits, psychological stress, comorbid conditions, and even the composition of the oral microbiome. Notably, psychological factors play a crucial role; some researchers have pointed out that elevated cortisol levels in anxious patients significantly affect the NLR value (Rahimi et al., 2024), which may interfere with the functional performance of neutrophils and obscure their true role. Therefore, future efforts should focus on reducing the use of subjective criteria and standardizing inclusion criteria to enhance reproducibility across studies, thereby providing valuable insights for the clinical application of neutrophil-related indicators. Moreover, the heterogeneity of neutrophils plays a crucial role in the progression of OPMDs to oral cancer. The functional transition from pro-inflammatory to pro-cancer states, alongside the differentiation from immature to immunosuppressive subpopulations, collectively contributes to the complexity of disease progression. By focusing on neutrophils as a critical point of investigation and employing technologies such as single-cell multi-omics and spatial transcriptomics, we aim to elucidate the changes that neutrophils undergo in oral mucosal diseases, their roles in the pathological processes, and the factors that guide neutrophils in assuming specific roles or functions. This understanding will enhance our knowledge of the pathogenesis of oral mucosal diseases, ultimately facilitating early intervention and personalized treatment for these conditions.
